# A Formalin‐Inactivated Vaccine Enhances Survival and Mitigates Horizontal Transmission of Red Sea Bream Iridovirus (RSIV) in Rock Bream ( *Oplegnathus fasciatus* ): Insights From Viability Quantitative PCR

**DOI:** 10.1111/jfd.70176

**Published:** 2026-03-26

**Authors:** Sung‐Bin Moon, Gyoungsik Kang, HyeongJin Roh, Yoonhang Lee, Min‐Jae Kim, Min‐Young Sohn, Ha‐Jeong Son, Chan‐Il Park, Kyung‐Ho Kim

**Affiliations:** ^1^ Department of Aquatic Life Medicine, College of Marine Sciences Gyeongsang National University Tongyeong Republic of Korea; ^2^ Department of Aqualife Medicine Chonnam National University Yeosu Republic of Korea; ^3^ Aquatic Disease Control Division National Fishery Products Quality Management Service Busan Republic of Korea; ^4^ Department of Marine Biology and Aquaculture, College of Marine Sciences Gyeongsang National University Tongyeong Republic of Korea

**Keywords:** horizontal transmission, infectious virions, red sea bream iridovirus, vaccine efficacy, viability qPCR

## Abstract

Formalin‐inactivated vaccines are widely employed as a primary preventive strategy against red sea bream iridoviral disease (RSIVD), which poses a substantial economic threat to the aquaculture of rock bream ( *Oplegnathus fasciatus* ). However, conventional quantitative PCR (qPCR) cannot differentiate infectious virions from noninfectious vaccine residues or viral debris, limiting accurate assessment of vaccine efficacy. Therefore, the present study aimed to evaluate the protective efficacy and viral shedding dynamics of a formalin‐inactivated vaccine using propidium monoazide (PMAxx)‐based viability qPCR (vqPCR). Vaccination demonstrated robust protection, achieving a relative percent survival of approximately 80% in immersion challenges and significantly reducing viral shedding into seawater. Notably, comparative analysis revealed that conventional qPCR significantly overestimated viral risk in vaccinated fish by detecting noninfectious DNA artifacts. Although vaccination did not confer sterilizing immunity, it suppressed infectious viral replication to sublethal levels, effectively preventing horizontal transmission to naïve cohabitants. Furthermore, disease progression and shedding kinetics were temperature‐dependent, occurring more rapidly at 25°C than at 20°C. Overall, these findings highlight that vaccination induces functional sterilization of shedding and underscore the need to adopt vqPCR for accurate epidemiological risk assessment in aquaculture populations.

## 1 Introduction

Red sea bream iridoviral disease (RSIVD) is caused by infection with *Megalocytivirus pagrus1* (subfamily Megalocytivirinae, family Iridoviridae), which has caused mass mortality and substantial economic losses in rock bream ( *Oplegnathus fasciatus* ) aquaculture in Korea (Jeong et al. 2006; Kim et al. 2023a). First reported identified in Japan in the 1990s (Inouye et al. 1992), RSIVD has since become endemic to East Asia, posing persistent and severe damage to the marine aquaculture industry (Kurita and Nakajima 2012). Specifically, in Korea, the disease was first reported in cultured red sea bream ( *Pagrus major* ) in 1998 and has since emerged as one of the most prevalent and devastating diseases affecting rock bream production (Sohn et al. 2000; Kim et al. 2002). Based on sequence analysis of the major capsid protein and adenosine triphosphatase genes, the causative agent has been classified into three distinct genotypes: red sea bream iridovirus (RSIV), infectious spleen and kidney necrosis virus, and turbot reddish body iridovirus (Nakajima and Kunita 2005).

RSIV infects a range of susceptible fish species, including rock bream, red sea bream, flathead grey mullet fish ( *Mugil cephalus* ), and Asian sea bass ( *Lates calcarifer* ) (Kurita and Nakajima 2012; WOAH 2021). The virus exhibits high tropism for haematopoietic organs, most notably the spleen and kidney, where active viral replication occurs (Sumithra et al. 2022; Kim et al. 2023a, 2024a). Viral replication strongly depends on temperature; at water temperatures exceeding 25°C, the virus undergoes accelerated proliferation within host tissues (Kim et al. 2024a). Notably, the extent of viral replication within the host is positively correlated with the degree of viral shedding into the surrounding aquatic environment (Kim et al. 2024a). Consequently, elevated temperatures drive both rapid viral amplification and increased shedding, enhancing the efficiency of horizontal transmission. This temperature‐dependent synergy contributes to disease progression and high mortality and potentially underlies the severe outbreaks consistently observed during the summer months in Korea and Japan (Kawato et al. 2021; National Fishery Products Quality Management Service 2023). To mitigate these losses, vaccination has emerged as a key and widespread strategy (Nakajima et al. 1999).

In high‐density aquaculture systems, formalin‐inactivated vaccines are widely used owing to their cost‐effectiveness and ease of administration (Min et al. 2024). Various RSIV vaccines have been developed in Japan (Nakajima et al. 1999; Caipang et al. 2006) and Korea (Kwon et al. 2020a; Min et al. 2024), and their efficacy has been predominantly validated based on the relative percent survival (RPS) as the primary metric. For example, Min et al. (2024) reported that the RSIV genotype II vaccine achieved an RPS of approximately 40%–80% under laboratory and field conditions. However, a more accurate assessment of vaccine efficacy requires experimental designs that mimic natural infection routes. For example, Kwon et al. (2020a) employed a cohabitation challenge model to verify vaccine efficacy, enabling a more precise protection assessment. However, this approach has limitations in characterizing pathogen dynamics through waterborne horizontal transmission. Consequently, the broader epidemiological impact of vaccination in real‐world aquaculture settings, particularly its effect on viral shedding kinetics and the transmission potential of vaccinated survivors, remains largely unexplored.

Evaluating vaccine efficacy is also considerably limited by the inability of traditional diagnostic tools to distinguish infectious virions from noninfectious viral DNA residues. For example, conventional quantitative PCR (qPCR) detects total viral nucleic acids regardless of infectivity, potentially overestimating viral burden and transmission risk in vaccinated fish (Mohr et al. 2015; Kim, Kang, et al. 2024b). This limitation can be addressed by the application of viability quantitative PCR (vqPCR) using propidium monoazide (PMAxx). Upon photoactivation, PMAxx selectively penetrates compromised capsids, such as those from formalin‐inactivated or heat‐killed viruses, and covalently binds to their DNA, preventing amplification during PCR (Parshionikar et al. 2010; Randazzo et al. 2016). Complementing this approach, the recent development of iron flocculation‐based quantification methods has enabled the investigation of viral dynamics in seawater (Kawato et al. 2016). Using this technique, epidemiological investigations of RSIV in seawater have been conducted across various environmental conditions (Kawato et al. 2021; Kim et al. 2023b). Furthermore, this method has been successfully adapted for the detection of various aquatic viral diseases (Kim et al. 2023b; Shin et al. 2025). Notably, combining FeCl_3_‐based iron flocculation with viability qPCR has demonstrated superior performance in distinguishing infectious RSIV from seawater samples (Kim et al. 2023b). Applying these advanced diagnostic techniques to vaccine evaluation enables a direct comparison of infectious viral loads in both host tissues and the rearing environment, allowing precise assessment of actual viral replication dynamics and the true reduction in infectivity following vaccination. This integrated approach ultimately provides deep insights into the broader impact of vaccination on field transmission dynamics and disease epidemiology.

In the present study, we comprehensively investigated the impact of vaccination on horizontal transmission and RSIV dynamics within the aquatic environment using a combined approach of PMAxx‐based viability qPCR and iron flocculation concentration. We performed comparative analyses of viral viability in host tissues and rearing water under different infection routes (intraperitoneal [IP] vs. immersion [IM]) and water temperatures (20°C and 25°C). This study aimed to elucidate the mechanisms by which vaccination influences RSIVD epidemiology and provide key insights for establishing effective vaccination‐based risk management strategies in rock bream aquaculture.

## 2 Materials and Methods

### 2.1 Virus and Cell Line Culture

The RSIV genotype II (accession number: AY532608), isolated from rock bream in Korea (Kim et al. 2022), was used in this study. For high‐titre virus propagation, the virus was cultured in the *Pagrus major* fin (PMF) cell line following a previously described protocol with minor modifications (Kwon et al. 2020b). Briefly, PMF cells were cultured at 25°C in L‐15 medium (Gibco, Thermo Fisher Scientific, Waltham, MA, USA) supplemented with 10% fetal bovine serum (FBS; Gibco) and 1% antibiotic‐antimycotic (Gibco) in T‐75 cell flasks. Cells were then washed with Dulbecco's phosphate‐buffered saline, after which the virus was inoculated into L‐15 medium containing 2% FBS and 1% antibiotic‐antimycotic. Upon confirmation of cytopathic effects, the culture supernatant was collected and passed through a 0.45 μm syringe filter to remove cellular debris. Freshly prepared viruses were used for all experiments on the same day. Viral titre of RSIV was determined as described in Section 2.7.

### 2.2 Experimental Fish

The rock bream used in this study (13.94 ± 4.07 cm in length, 11.58 ± 3.33 g in weight) was purchased from a private fish farm located in Geoje‐si, Gyeongsangnam‐do, Republic of Korea. Upon arrival at the experimental facility, the fish were evenly allocated to four 450‐L circular flow‐through tanks at a density of 550 individuals per tank and acclimated for 2 weeks before the experiment. Before vaccination and viral challenge, five fish from each tank were randomly selected and screened for RSIV infection using qPCR to confirm the absence of pre‐existing infection; this experiment is detailed below. Throughout the two‐week acclimation period preceding vaccination and infection, water temperature was maintained at 25°C ± 1°C. All animal experiments were approved by the Institutional Animal Care and Use Committee of Gyeongsang National University (approval number: GNU‐241204‐E0241).

### 2.3 Vaccine Preparation

The formalin‐inactivated RSIV vaccine used in this study was prepared as previously described (Kwon et al. 2020a). Vaccination was conducted on fish acclimated at 25°C following the manufacturer's recommendations. Final concentration of vaccine was prepared as 10^10^ copies/fish.

### 2.4 Experiment 1: IP Injection Challenge

**FIGURE 1:**
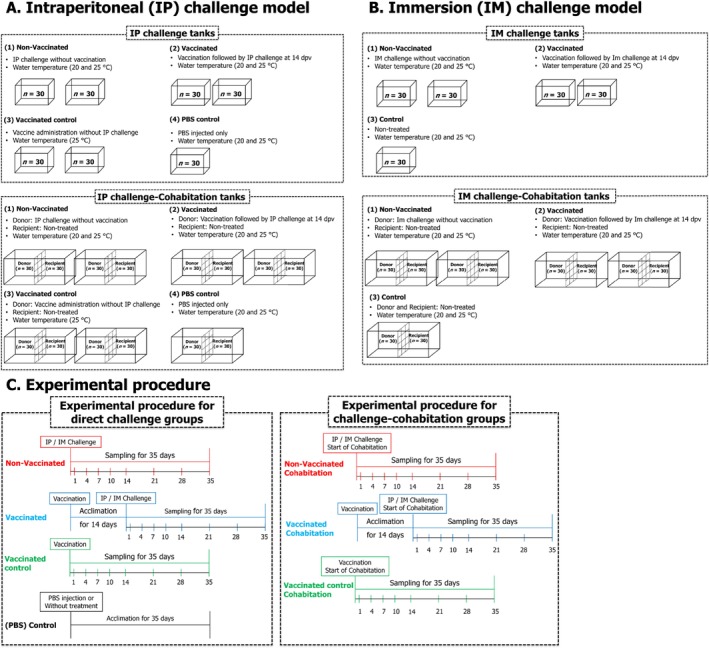
Comprehensive schematic illustration of the experimental strategy for evaluating the efficacy of vaccines against red sea bream iridovirus (RSIV) across different infection routes. Schematic diagram of experimental groups classified by infection route and vaccination status: (A) Intraperitoneal (IP) injection and (B) immersion (IM) challenge models. (C) Experimental timeline of vaccination and infection protocols based on housing configuration (isolated vs. cohabitation). Monitoring of viral titers in tissues and rearing seawater at the indicated time points. DPV, days post‐vaccination.

To assess the protective efficacy of the vaccine in a controlled artificial infection model, fish were subjected to an IP challenge. The experimental design and timeline are illustrated in Figure 1A,C, respectively. After preparing the virus and vaccine, RSIV infection was performed via IP injection, with each fish receiving 10^6^ infectious copies. Similarly, vaccination was administered via IP at a dose of 100 μL per fish. Throughout the experiment, all tanks were maintained as static systems with a daily 50% water exchange.

For every treatment group in the rearing configurations described below, duplicate tanks were established: one for periodic tissue sampling (viral load and viability) and the other for monitoring daily mortality, prevalence rate and periodic seawater viral shedding (viral load and viability) over 35 days.

#### 2.4.1 IP Injection Challenge

In this configuration, the challenge model was designed to assess the protective efficacy of the vaccine under controlled experimental infection conditions. Treated rock bream (*n* = 30) were maintained in 50‐L tanks to assess vaccine efficacy at the individual level. The experiment consisted of four groups: non‐vaccinated (intraperitoneal challenge), vaccinated (vaccination followed by intraperitoneal challenge at 14 d post‐vaccination [dpv]), vaccinated control (vaccine only, without challenge), and phosphate‐buffered saline (PBS) control (PBS injection only). Trials were conducted at two water temperatures (20°C and 25°C), except for the vaccinated control group, which was tested only at 25°C.

#### 2.4.2 IP Injection–Cohabitation (IP‐Co) Challenge

To assess the risk of horizontal transmission from artificially infected rock bream, cohabitation trials were conducted using 100‐L tanks divided into compartments by a mesh partition (3 × 3 cm openings). Treated rock bream (Donors, *n* = 30) were introduced into one compartment, whereas naïve rock bream (Recipients, *n* = 30) were placed in the adjacent compartment. The donor groups corresponded to the treatment groups: non‐vaccinated, vaccinated, vaccinated control, and PBS control donors. Trials were also conducted at 20°C and 25°C, except for the vaccinated control group (only at 25°C).

### 2.5 Experiment 2: IM Challenge

An IM challenge was performed to simulate natural infection routes in aquaculture. The experimental design and timeline are illustrated in Figure 1B,C, respectively. Vaccination was performed as described in Section 2.4, whereas RSIV infection was performed via immersion. Specifically, fish were exposed to RSIV at a concentration of 10^6^ copies/L in 200 L of seawater for 6 h at 25°C and 20°C. Following exposure, fish were transferred to fresh seawater tanks. Housing conditions, water management, and tank replication strategies (sampling vs. mortality) were identical to those in Experiment 1.

#### 2.5.1 IM Challenge

To evaluate the protective efficacy of the vaccine against immersion‐based infection, fish were subjected to a direct IM challenge simulating waterborne infection. Following the IM exposure, fish (*n* = 30) were transferred to 50‐L tanks to assess the efficacy of the vaccine against waterborne infection at the individual level. The experiment consisted of three groups: non‐vaccinated (IM challenge without vaccination), vaccinated (vaccination followed by IM challenge at 14 dpv) and control (non‐treated). The experiments were conducted at both 20°C and 25°C, except for the control group, which was maintained only at 25°C.

#### 2.5.2 IM–Cohabitation Challenge

To evaluate the transmission potential of immersion‐exposed fish, a cohabitation model was established in 100‐L tanks divided by a mesh partition with 3 × 3 cm openings. Donor fish (*n* = 30), previously exposed to the virus via IM, were placed in one compartment, whereas naïve recipients (*n* = 30) were placed in the adjacent compartment. The donor groups included non‐vaccinated donors, vaccinated donors and controls (non‐treated).

### 2.6 Sampling Procedures and Efficacy Evaluation

Changes in viral load and viability in the spleen were assessed at 1, 4, 7, 10, 14, 21, 28 and 35 dpv for the vaccination‐only group and at 1, 4, 7, 10, 14, 21, 28 and 35 d post‐infection (dpi) (Figure 1C). At each time point, three fish were randomly sampled from each treatment group, including the direct challenge group and donors and recipients from the cohabitation groups. Seawater samples (500 mL) were collected from each treatment group at the same point for viral load analysis.

Survival rates were monitored and recorded daily for 35 d after the last treatment (vaccination or infection) in each group. When mortality occurred, deceased fish were immediately collected. To determine prevalence, all the surviving fish from the tanks designated for mortality monitoring and seawater viral copy assessment were sampled at the end of the experiment. Protective efficacy, defined as RPS, was calculated as follows: RPS (%) = [1 − (mortality rate in the treated group/mortality rate in the control group)] × 100.

### 2.7 Quantification of Viral Loads and Viability Analysis

#### 2.7.1 DNA Extraction From Fish Tissue and PMAxx Based Viability qPCR

Viral load and viability were assessed in tissue and seawater samples using qPCR and PMAxx‐based viability qPCR, providing an estimate of viral genome copies and the proportion of intact viruses, respectively. To assess viral load and viability in fish tissues, three fish were randomly selected from each treatment group and euthanized using benzocaine (Sigma‐Aldrich, St. Louis, MO, USA). Spleens were aseptically excised, and approximately 20–50 mg of splenic tissue was homogenized in 1 mL of PBS. The homogenate was centrifuged at 10,000 × *g* for 10 min at 4°C, and the supernatant was filtered through a 0.45 μm syringe filter. The filtered supernatant was then divided into two replicates of 200 μL each in 1.5 mL tubes for subsequent viral load and viability analyses.

For viral load quantification, DNA was extracted from one 200 μL aliquot using a Genomic DNA Extraction Kit (Bioneer, Daejeon, Republic of Korea) following the manufacturer's protocol. The extract was used as a template for qPCR.

For viability assessment, PMAxx‐based viability qPCR was performed as described by Kim et al. (2023b), with some modifications. Briefly, PMAxx (Biotium, Fremont, CA, USA) was added to the second aliquot to a final concentration of 50 μM, and the sample was incubated in the dark for 30 min. The reaction mixture was then exposed to LED light for 30 min using a photoactivation system (PMA‐Lite LED Photolysis Device; Biotium) to activate PMAxx. Subsequently, DNA was extracted using a Genomic DNA Extraction Kit (Bioneer) following the manufacturer's protocol.

#### 2.7.2 Viral Concentration Determination in Seawater via Iron Flocculation

To monitor viral load and viability in seawater, viruses were concentrated from seawater samples using the iron‐based flocculation method, developed by Kawato et al. (2016) and Kim et al. (2024a). At the same time points as tissue sampling, 500 mL of seawater was collected from each tank and pre‐filtered through a Whatman glass microfiber grade GF/A filter (Cytiva, Marlborough, MA, USA). FeCl_3_ solution (50 μL; 4.83 g FeCl_3_·6H_2_O in 100 mL of distilled water) was added to the filtered seawater, and the mixture was stirred using a magnetic stirrer for 1 h. Virus–iron flocs were captured on a nucleopore track‐etch membrane (pore size 0.8 μm). The membrane was then transferred to a 5 mL round‐bottom tube with 1.5 mL of oxalate‐EDTA elution buffer, prepared as previously described (Kim et al. 2022), and incubated at 4°C in the dark for 18 h to recover a 1.5 mL viral suspension. The recovered viral suspension was aliquoted into six 1.5 mL tubes, each containing 200 μL, providing three replicates for standard qPCR (viral load) and three replicates for viability qPCR. DNA extraction was performed as described in Section 2.7 Ministry of Education of the Republic of Korea for both the untreated and PMAxx‐treated samples.

#### 2.7.3 Real‐Time qPCR Amplification Conditions

All extracted DNA samples from spleen or seawater were used for RSIV detection via qPCR as previously described (Kim et al. 2022). The reaction mixture (25 μL) consisted of 12.5 μL of HS Prime qPCR Premix (2×) (Genet Bio Co. Ltd., Daejeon, Republic of Korea), 900 nM of each forward and reverse primer, 250 nM of TaqMan probe, and 5 μL of template DNA, either untreated or PMAxx‐treated. The qPCR cycling conditions were as follows: initial denaturation at 95°C for 1 min, followed by 45 cycles of 95°C for 10 s and 60°C for 20 s. All the qPCR assays were performed using the Thermal Cycler Dice Real Time System III TP950 (Takara Bio, Shiga, Japan). The viral concentration in seawater was normalized to the biomass of the surviving fish (g) and expressed as the viral shedding ratio, reported as viral copies/L per gram of surviving fish biomass (copies·L^−1^·g^−1^).

### 2.8 Statistical Analysis

Viral copy numbers were log‐transformed (Log_10_) before analysis to approximate a normal distribution. The correlation between the total and infectious viral loads was analysed using linear regression analysis. Differences in regression slopes were compared using analysis of covariance to compare viral replication efficiency among groups. Survival curves were analysed using the Kaplan–Meier method. Differences in splenic tissue viral titers based on mortality status and detection methods (conventional qPCR vs. vqPCR) were analysed using one‐way analysis of variance with Dunnett's test, while intragroup differences were assessed using the log‐rank (Mantel–Cox) test. Statistical significance was considered at *p* < 0.05 (*p < 0.05, **p < 0.01, ***p < 0.001, ****p < 0.0001).

## 3 Results

### 3.1 Experiment 1: IP Injection Challenge

#### 3.1.1 Direct IP Challenge

**FIGURE 2:**
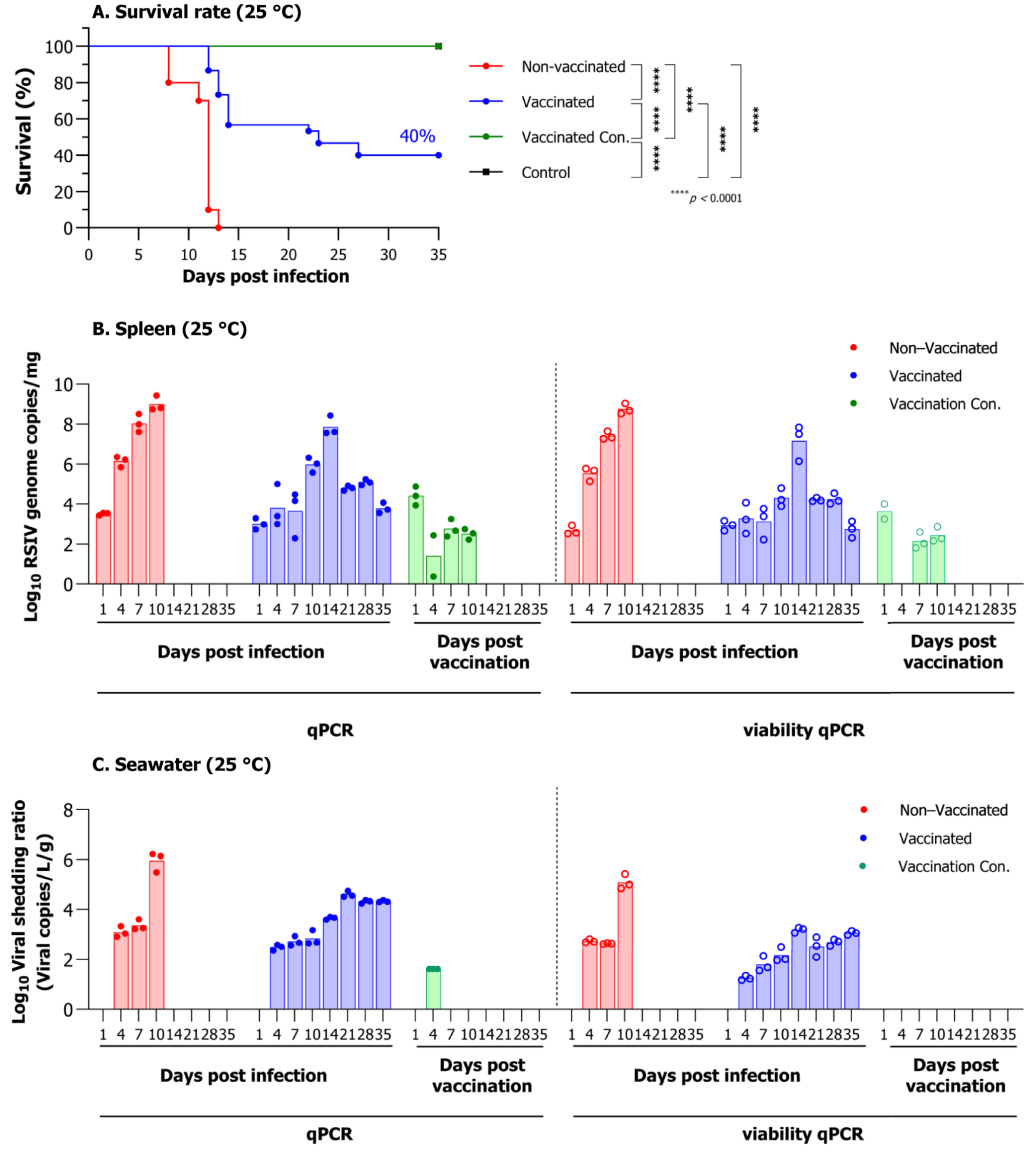
Evaluation of protective efficacy and viral replication kinetics in rock bream following intraperitoneal (IP) red sea bream iridovirus (RSIV) challenge at 25°C. (A) Kaplan–Meier cumulative survival curves of fish throughout 35 days following infection. (B) Temporal analysis of total genomic DNA (qPCR) versus infectious viral titers (PMAxx‐based vqPCR) in the spleen to compare viral replication dynamics. (C) Quantification of viral copies released into the rearing water over time to assess shedding kinetics. Viral loads are expressed as Log_10_ copies/mg for tissue and Log_10_ copies/L/g fish biomass for seawater.

**FIGURE 3:**
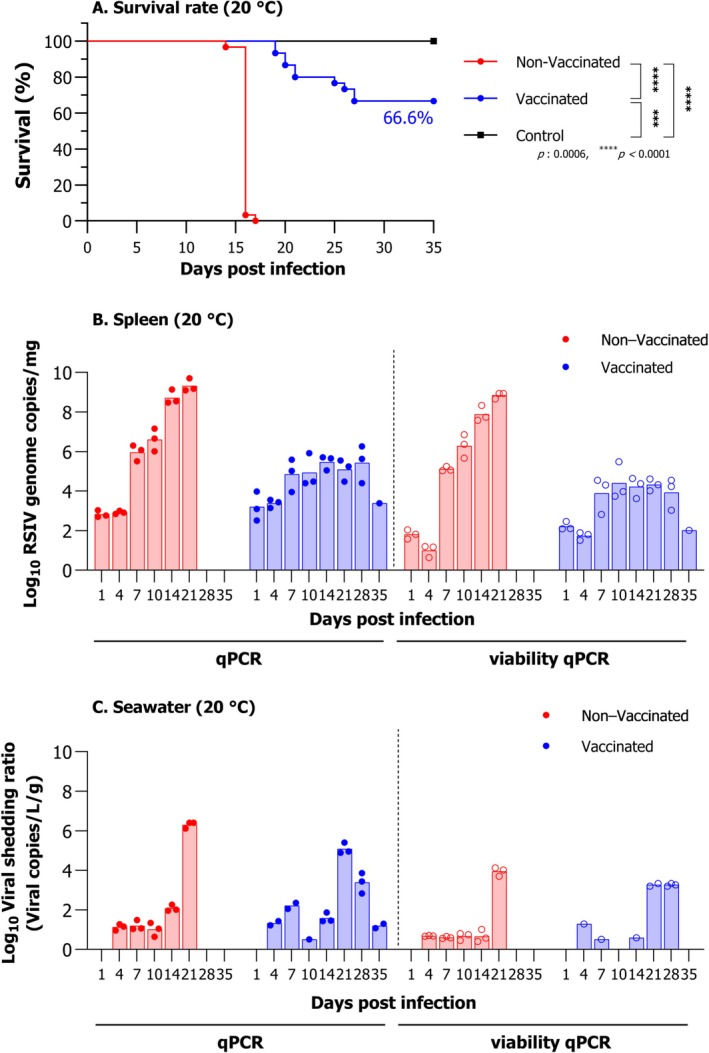
Evaluation of protective efficacy and viral replication kinetics in rock bream following intraperitoneal (IP) red sea bream iridovirus (RSIV) challenge at 20°C. (A) Kaplan–Meier cumulative survival curves of fish throughout 35 d following infection. (B) Temporal analysis of total (qPCR) versus infectious (vqPCR) viral loads in the spleen to compare viral replication dynamics. (C) Quantification of viral copies released into the rearing water over time to assess shedding kinetics.

Following IP challenge, non‐vaccinated fish experienced severe, temperature‐dependent mortality. At 25°C, rapid viral replication drove cumulative mortality to 100% by 13 dpi (Figure 2A). Although disease progression was slower at 20°C than at 25°C, cumulative mortality ultimately reached 100% by 17 dpi (Figure 3A). In contrast, vaccination significantly enhanced survival rates. Vaccinated fish exhibited significantly higher survival than non‐vaccinated controls, achieving RPS values of 40% at 25°C (Figure 2A) and 66.7% at 20°C (Figure 3A). Notably, no mortality occurred in the vaccinated control groups (non‐challenged), confirming the safety of the vaccine (Figure 2A).

Water temperature also strongly influenced RSIV infection kinetics. In the non‐vaccinated isolated challenge group, viral replication proceeded rapidly and lethally, resulting in 100% mortality. This was accompanied by a sharp spike in total viral copies within the spleen, peaking at approximately 10^8.9^ copies/mg (Figures 2B and 3B). Infectious viral copies, quantified using PMAxx‐based vqPCR, exhibited a similar trajectory, confirming that most of the detected viruses remained viable. Viral shedding into seawater closely paralleled tissue viral loads, peaking at 10^5^.^9^–10^6^.^1^ copies/L/g (Figures 2C and 3C). In contrast, the vaccinated fish exhibited delayed peak viral loads (10^7.8^–10^8.2^), which declined to less than 10^6^ copies/mg by 35 dpi (Figures 2B and 3B). Correspondingly, vaccinated fish demonstrated lower viral shedding than non‐vaccinated fish (Figures 2C and 3C). Notably, neither viral replication nor mortality was observed in the vaccinated control groups (non‐challenged), confirming vaccine safety (Figure 2B).

#### 3.1.2 IP Injection–Cohabitation Challenge

**FIGURE 4:**
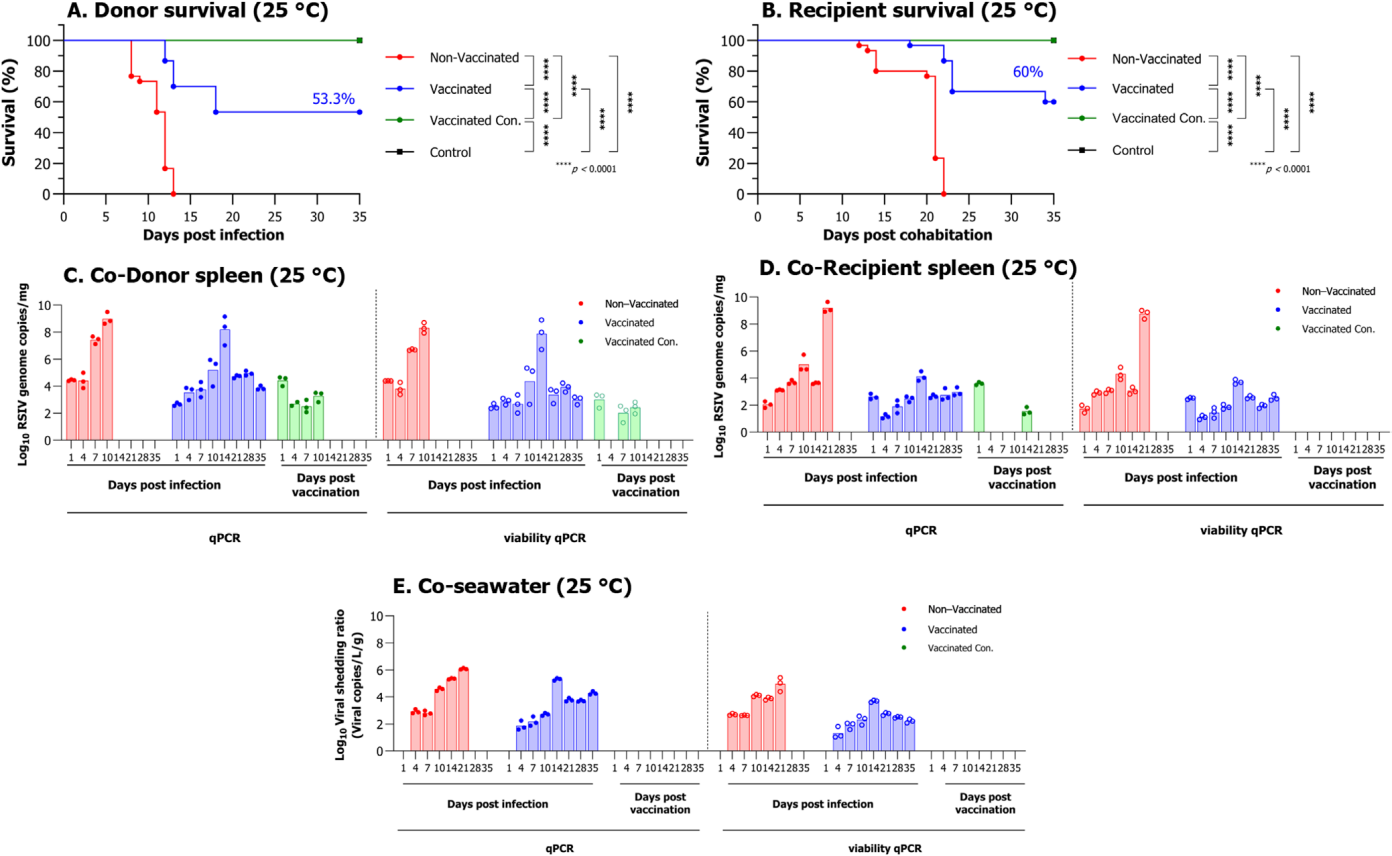
Horizontal transmission dynamics from intraperitoneal (IP)‐infected donors to naïve recipients at 25°C. (A, B) Cumulative survival rates of (A) donors and (B) cohabiting naïve recipients monitored for 35 d post‐infection. (C, D) Comparative analysis of total and infectious viral burdens in (C) donors and (D) recipients to evaluate the transmission of infection. (E) Monitoring of viral load dynamics in rearing water to assess exposure pressure.

**FIGURE 5:**
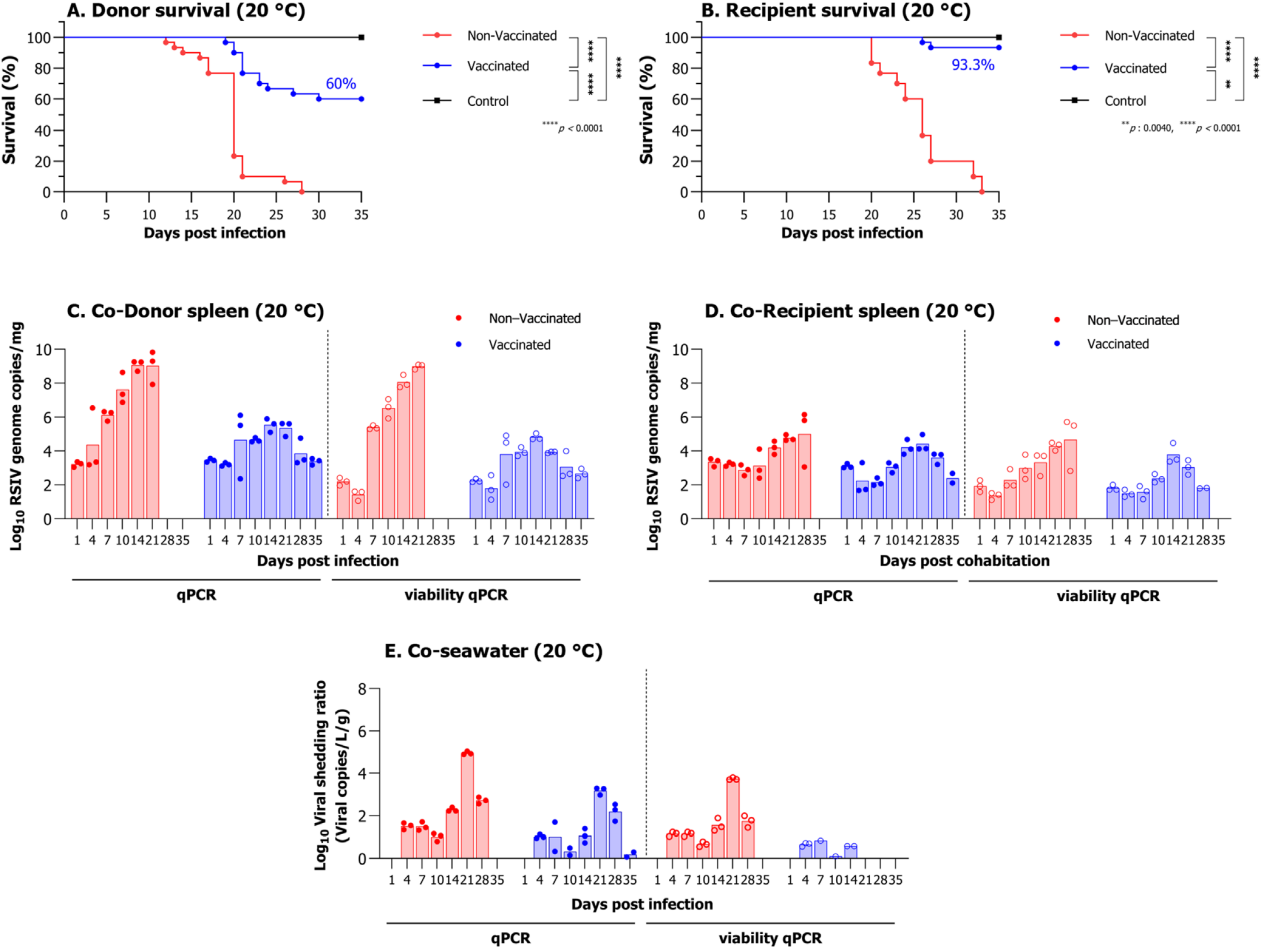
Horizontal transmission dynamics from intraperitoneal (IP)‐infected donors to naïve recipients at 20°C (A, B) Cumulative survival rates of (A) donors and (B) cohabiting naïve recipients. (C, D) Comparative analysis of total and infectious viral burdens in (C) donors and (D) recipients to evaluate the transmission of infection. (E) Monitoring of viral load dynamics in rearing water to assess exposure pressure.

We evaluated the risk of horizontal transmission by cohabiting naïve recipient fish with IP‐challenged donors. In the non‐vaccinated groups, donors efficiently transmitted the virus to recipients at both temperatures. Specifically, at 25°C, rapid viral shedding from donors led to 100% mortality in recipients by 22 dpi (Figure 4B), whereas complete mortality was achieved by 33 dpi at 20°C (Figure 5B). Notably, this transmission chain was significantly suppressed by vaccination of donor fish. In these vaccinated cohabitation groups, recipient survival was significantly higher than that in non‐vaccinated cohabitation groups (0% survival), with a survival rate of 60% at 25°C and 93.3% at 20°C. These results indicate that vaccination confers protection to individuals and strongly limits the horizontal spread of RSIV.

Recipient tissue viral loads closely mirrored those of donor fish, confirming successful waterborne transmission. Viral kinetics in the spleen of donor fish paralleled those observed in the isolated challenge groups. At 25°C, non‐vaccinated recipients exhibited rapid increases in viral load after 21 dpi (Figure 4D). In contrast, vaccinated recipients maintained splenic viral copies below 10^6^ copies/mg during 35 dpi at both temperatures (Figures 4D and 5D). Furthermore, vaccinated donors shed significantly lower amounts of the virus than non‐vaccinated individuals (Figures 4E and 5E), mitigating the viral challenge pressure on cohabiting recipients. Consistent with the challenge group, viral DNA was initially detected in the vaccinated control group post‐vaccination but became undetectable after 14 d. In recipients, viral DNA was detected via conventional qPCR on Days 1 and 14 after cohabitation; however, vqPCR analysis confirmed the absence of infectious viral particles in these fish (Figure 4D).

### 3.2 Experiment 2: IM Challenge

#### 3.2.1 IM Challenge

**FIGURE 6:**
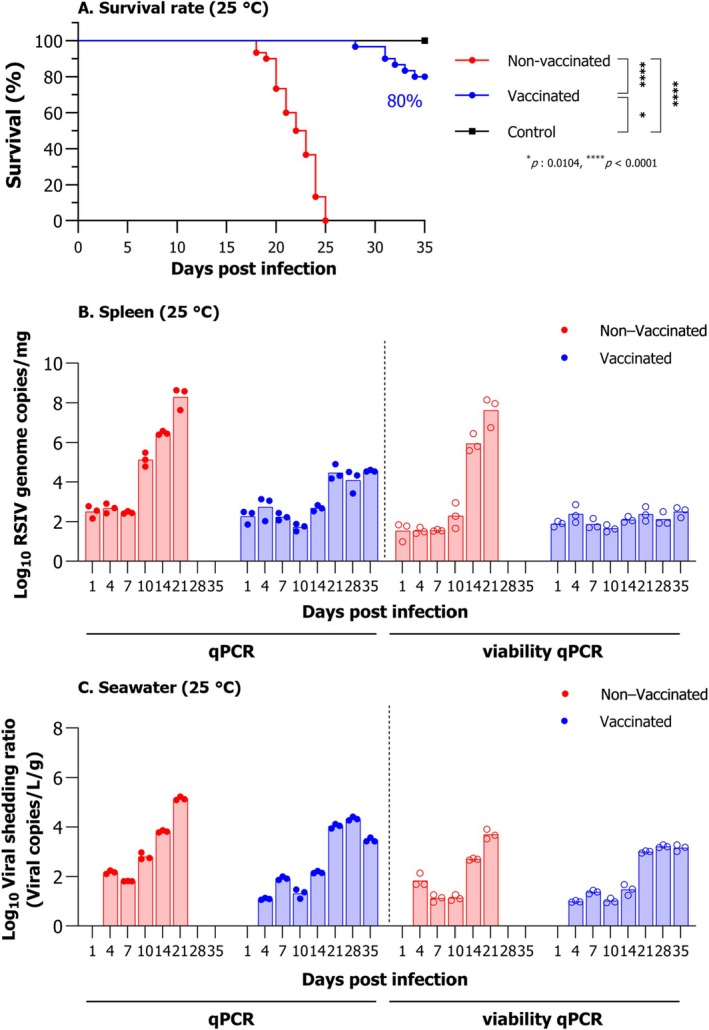
Protective efficacy and viral kinetics following immersion challenge (mimicking natural infection) at 25°C. (A) Cumulative survival rates monitored for 35 d post‐immersion. (B) Comparative quantification of total red sea bream iridovirus (RSIV) DNA (qPCR) and infectious virions (vqPCR) in the spleen to assess viral viability. (C) Temporal quantification of viral loads in rearing water to evaluate environmental shedding.

**FIGURE 7:**
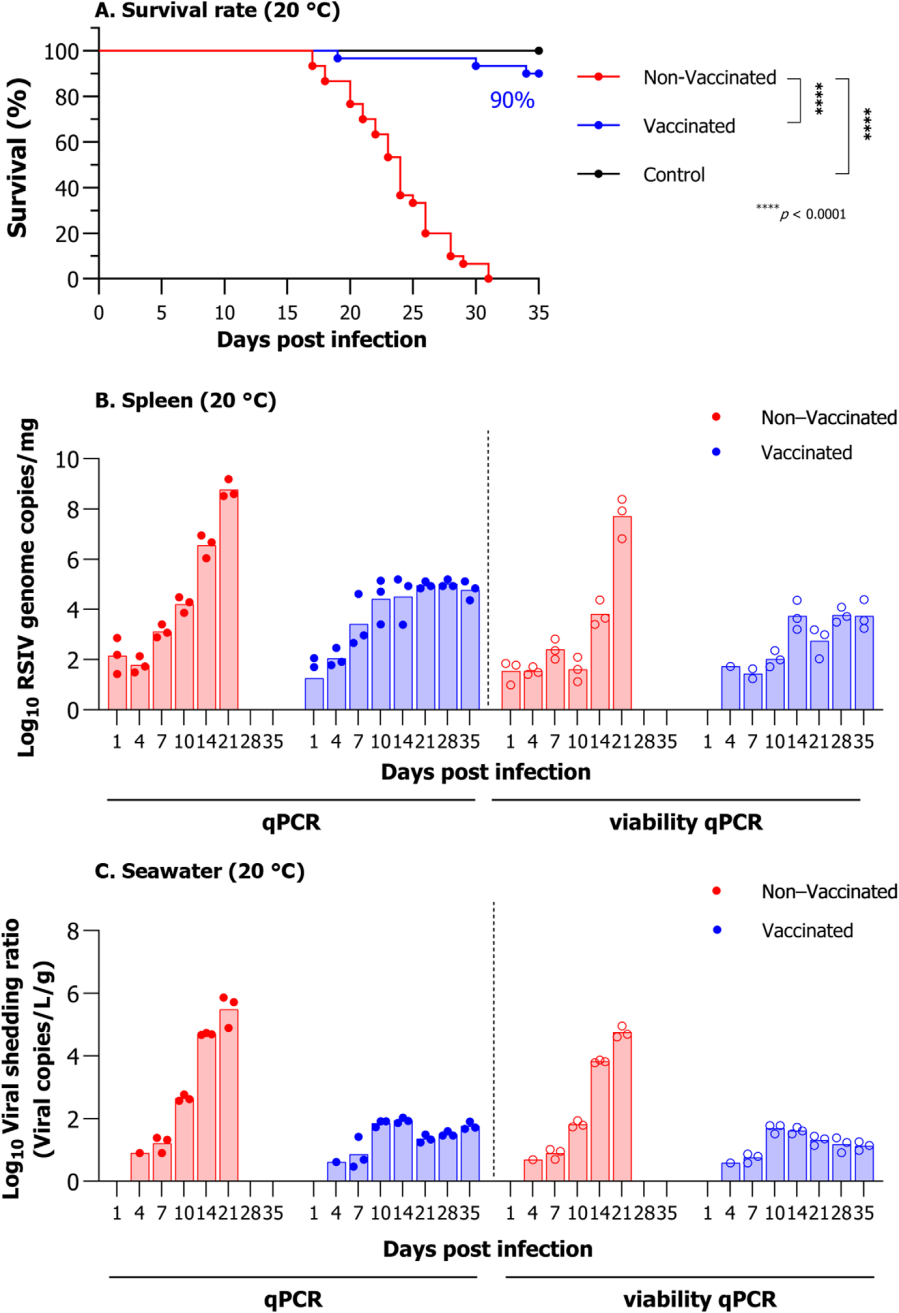
Protective efficacy and viral kinetics following immersion challenge (mimicking natural infection) at 20°C. (A) Cumulative survival rates monitored for 35 d post‐immersion. (B) Comparative quantification of total red sea bream iridovirus (RSIV) DNA (qPCR) and infectious virions (vqPCR) in the spleen to assess viral viability. (C) Temporal quantification of viral loads in rearing water to evaluate environmental shedding.

In the IM challenge designed to mimic natural infection routes, non‐vaccinated fish exhibited severe mortality regardless of water temperature. However, disease progression was faster at 25°C than at 20°C. with 100% cumulative mortality reached by 25 dpi (Figure 6A). At 20°C, the onset of mortality was relatively delayed, and cumulative mortality similarly reached 100% by the end of the experiment (Figure 7A). In contrast, vaccinated fish demonstrated superior protection in the IM model than in the IP‐challenged, as reflected by high survival rates with RPS values of 80% at 25°C (Figure 6A) and 90% at 20°C (Figure 7A). These results confirm the robust efficacy of the vaccine against waterborne exposure.

In non‐vaccinated fish, rapid mortality was accompanied by a dramatic surge in splenic viral loads (Figures 6B and 7B). This substantial tissue burden resulted in significant viral shedding into the surrounding seawater, with concentrations peaking at approximately 10^9^ copies at both 25°C (Figure 6C) and 20°C (Figure 7C). However, vaccination effectively suppressed viral replication. Throughout the experimental period, splenic viral loads in vaccinated fish remained significantly lower than those in non‐vaccinated controls (lower than 10^6^) (Figures 6B and 7B). Consequently, viral shedding kinetics were markedly attenuated, remaining orders of magnitude lower than those observed in the non‐vaccinated fish (Figures 6C and 7C). This potent suppression of viral replication prevented tissue viral load from reaching lethal thresholds in the host while simultaneously reducing environmental viral contamination.

#### 3.2.2 IM–Cohabitation Challenge

**FIGURE 8:**
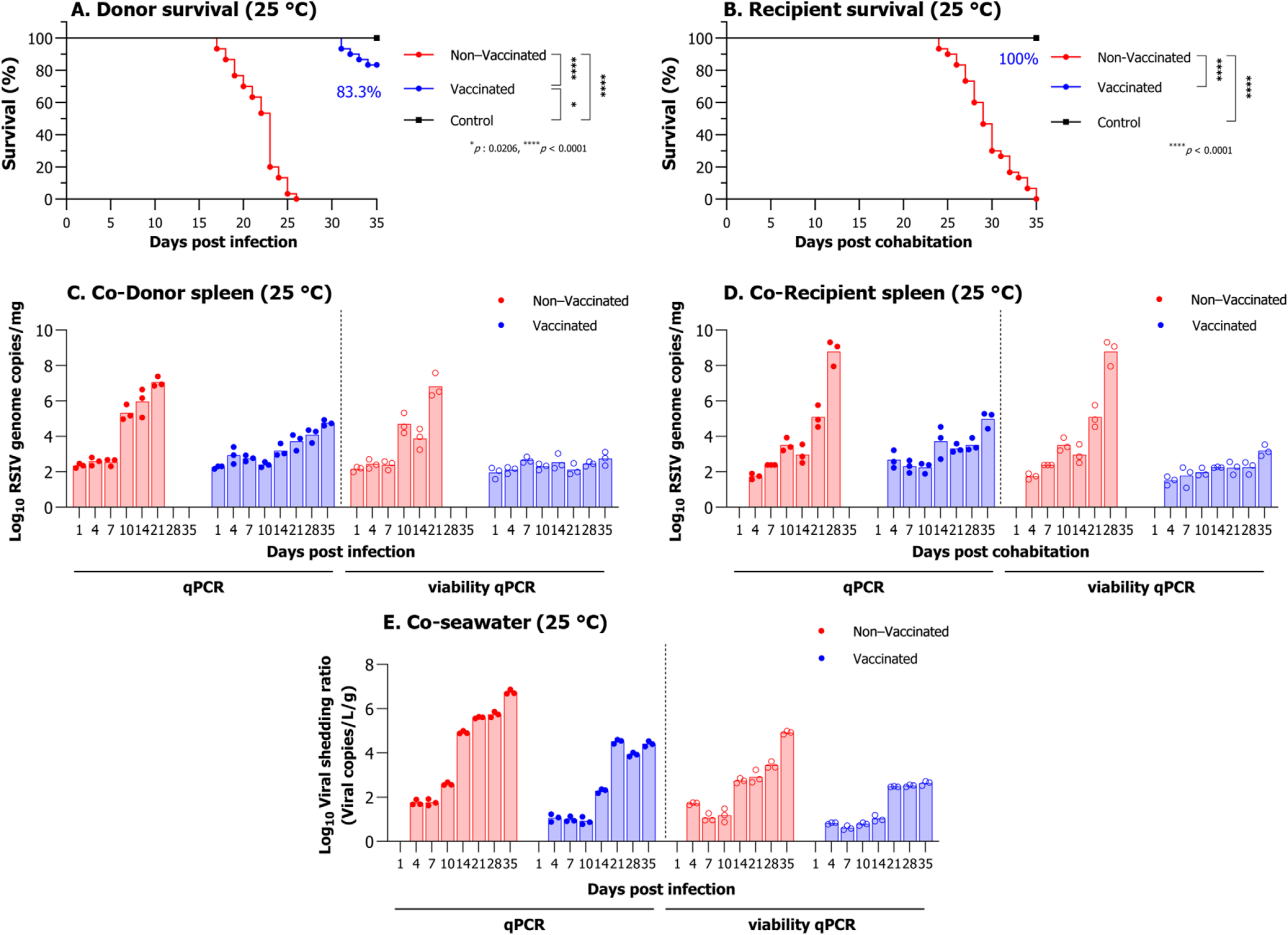
Assessment of viral shedding and horizontal transmission following immersion challenge at 25°C. (A, B) Cumulative survival rates of (A) donors and (B) naïve recipients in the cohabitation model. (C, D) Analysis of total and infectious viral loads in (C) donors and (D) recipients to identify transmission pathways. (E) Temporal quantification of viral loads in rearing water to evaluate environmental shedding.

**FIGURE 9:**
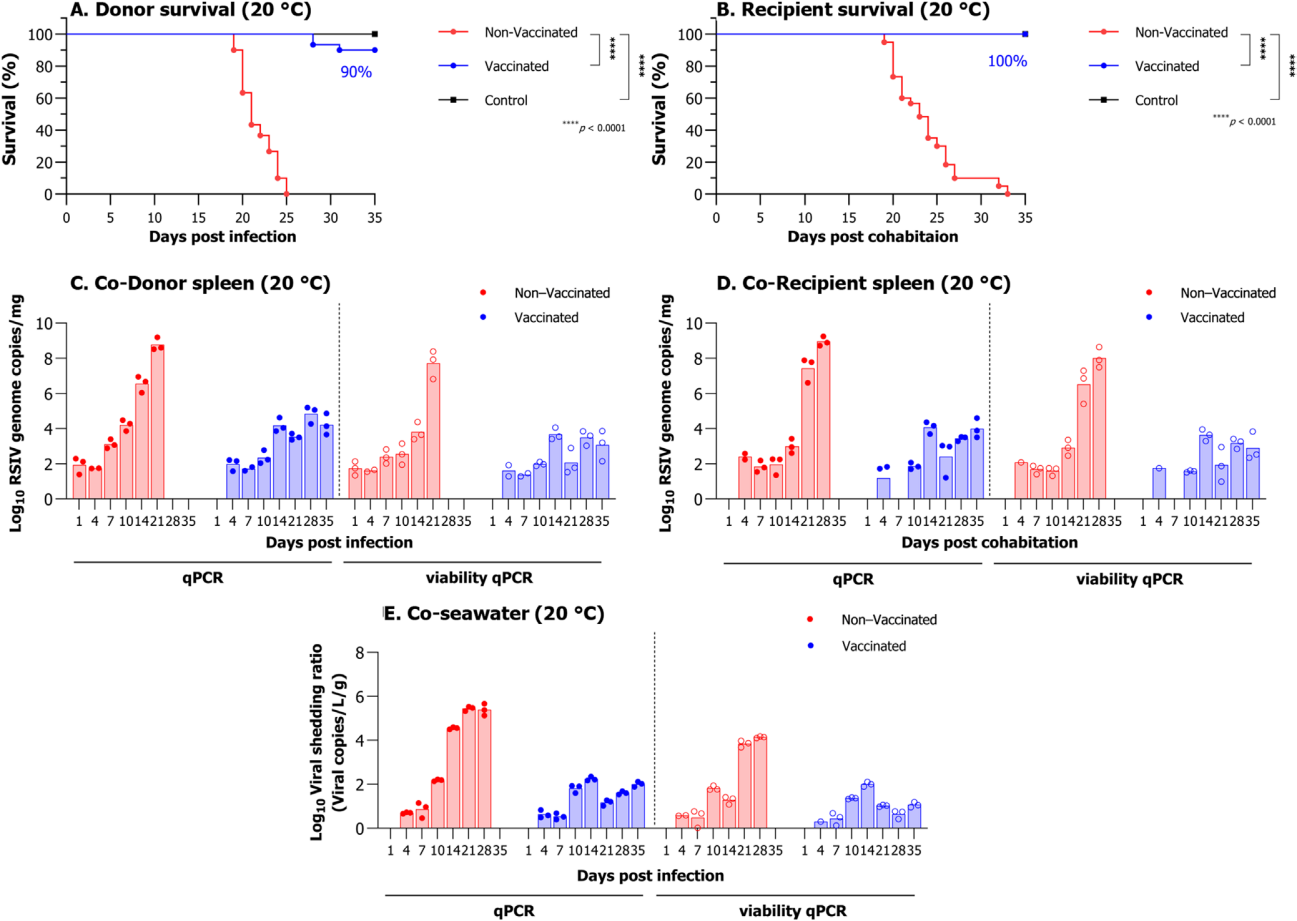
Assessment of viral shedding and horizontal transmission following immersion challenge at 20°C. (A, B) Cumulative survival rates of (A) donors and (B) naïve recipients in the cohabitation model. (C, D) Analysis of total and infectious viral loads in (C) donors and (D) recipients to identify transmission pathways. (E) Temporal quantification of viral loads in rearing water to evaluate environmental shedding.

The survival of naïve recipients was inextricably linked to disease progression in donor fish. In the non‐vaccinated groups, donors experienced severe mortality at both 25°C (Figure 8A) and 20°C (Figure 9A). This high donor mortality cohabiting naïve recipients to high infection pressure, resulting in 100% cumulative mortality by 33 dpi at 25°C (Figure 8B) and at 20°C by the end of the experiment (Figure 9B). In contrast, vaccination of the donors conferred robust protection, maintaining high donor survival rates at both temperatures (Figures 8A and 9A). Notably, this survival benefit extended to the cohabiting naïve fish, as no mortality occurred among recipients co‐housed with vaccinated donors (Figures 8B and 9B). These findings demonstrate that preventing disease in primary hosts (donors) effectively blocks lethal horizontal transmission to contact animals.

We then explored the mechanism underlying the protection of recipients by analysing viral kinetics and shedding. Non‐vaccinated donors exhibited exponential increases in splenic viral loads, peaking at high titers (Figures 8C and 9C). This extensive viral replication resulted in significant shedding of the virus into the rearing seawater (Figures 8E and 9E), exposing cohabiting recipients to high viral concentrations. Consequently, viral loads in the recipients co‐housed with non‐vaccinated donors surged in parallel with donor in parallel (Figures 8D and 9D). In contrast, vaccinated donors maintained significantly suppressed viral loads that remained below lethal thresholds throughout the study (Figures 8C and 9C). This suppression significantly reduced viral shedding into the water column, maintaining environmental viral loads at minimal or undetectable levels (Figures 8E and 9E). Consequently, recipients co‐housed with vaccinated donors exhibited no evidence of active infection, with splenic viral loads remaining negligible throughout the observation period (Figures 8D and 9D). These findings confirm that the vaccine interrupted the chain of transmission by limiting the shedding of infectious virions from the primary host.

### 3.3 Comparative Analysis of Infectivity Dynamics and Method‐Dependent Correlations

**FIGURE 10:**
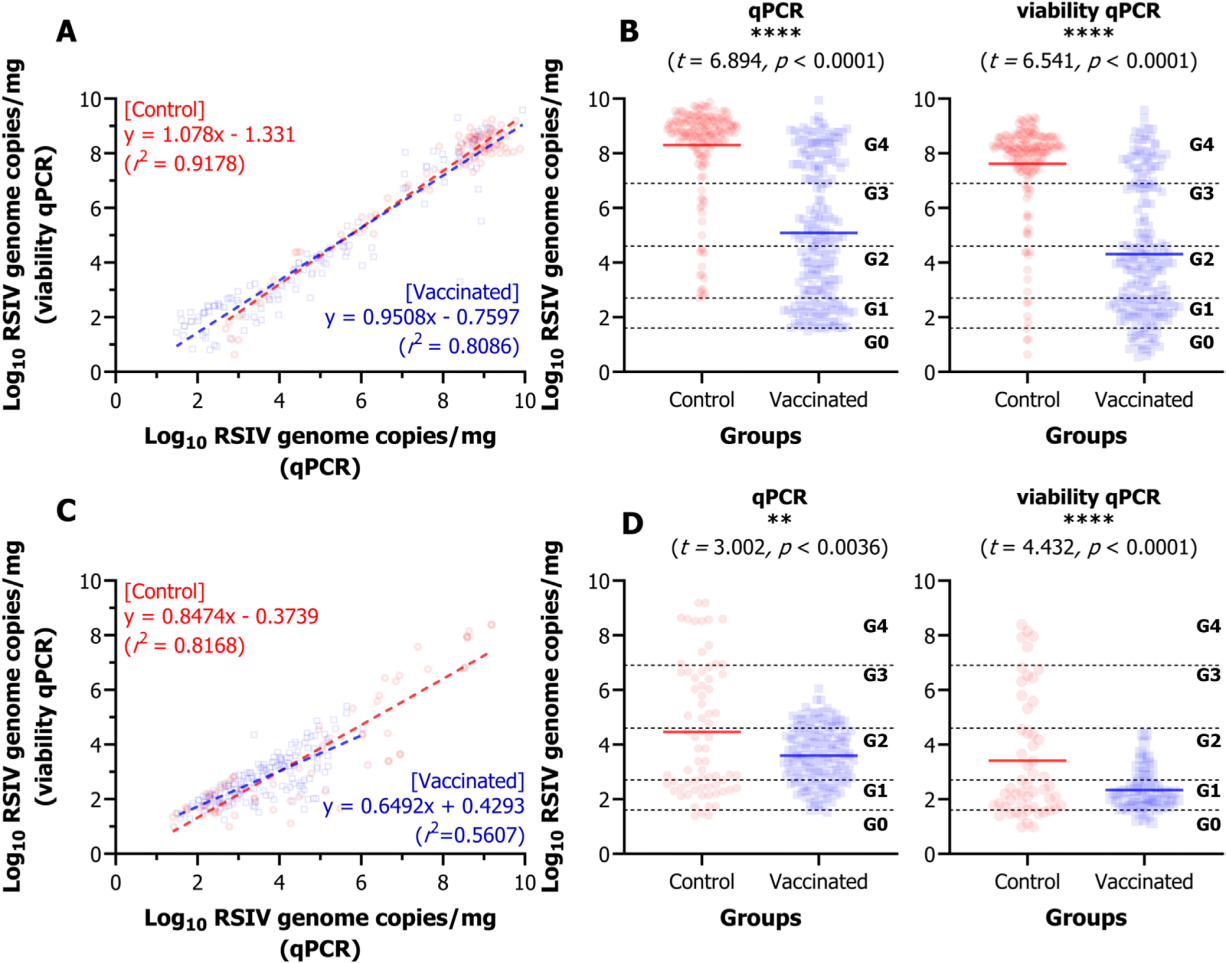
Route‐dependent dissociation between genomic load (qPCR) and infectious titers (vqPCR) in vaccinated rock bream individuals. (A, B) Intraperitoneal (IP) injection model; (C, D) Immersion challenge model. (A, C) Linear regression analysis: Scatter plots correlating total viral DNA (qPCR, *X*‐axis) with infectious titers (vqPCR, *Y*‐axis). Red and blue circles represent non‐vaccinated and vaccinated groups, respectively. Panel (C) depicts a weakened correlation and a significantly reduced regression slope in the vaccinated group under immersion conditions (C), highlighting the decoupling of viral quantity and quality. (B, D) Comparative quantification (Violin plots): Distribution of viral loads detected via qPCR versus vqPCR. The horizontal dotted lines indicate the thresholds for histopathological severity grades (G0–G4) based on viral copy numbers, as previously defined by Kim et al. (2024a): G0 (< 10^1.6^ copies/mg), G1 (10^1.6^–10^2.7^ copies/mg), G2 (10^2.7^–10^4.6^ copies/mg), G3 (10^4.6^–10^6.9^ copies/mg), and G4 (> 10^6.9^ copies/mg). Asterisks indicate significant differences between detection methods (**p < 0.01, ****p < 0.0001).

To elucidate the effect of vaccination on viral viability, we performed a comparative regression analysis between total viral DNA (assessed using conventional qPCR) and infectious virions (assessed using vqPCR) across different infection models (Figure 10). A global comparison revealed that infectious titers were consistently lower than total genomic copies across all experimental groups, confirming the widespread presence of noninfectious viral residues regardless of the infection route.

However, the correlation dynamics between genomic load and infectivity were significantly influenced by both infection route and vaccination status. In the intraperitoneal injection model (Figure 10A), both non‐vaccinated and vaccinated groups exhibited strong positive linear relationships (*R* ^2^ = 0.9178 and 0.8086, respectively). Notably, the regression slopes were statistically comparable (1.078 vs. 0.9508), indicating that when the virus circumvents physical barriers via direct injection, the ratio of infectious virions to total DNA remains relatively constant regardless of the vaccination status. Comparative quantification further confirmed that vqPCR values were significantly lower than conventional qPCR values in both groups (Figure 10B), reflecting the presence of non‐viable viral genetic material.

In contrast, the IM challenge model revealed a significant dissociation between viral DNA load and true infectivity in vaccinated individuals (Figure 10C). While the non‐vaccinated control group maintained a robust correlation between total viral DNA and infectious titre (*R* ^2^ = 0.8168) with a steep regression slope (0.8474), the vaccinated group exhibited a weakened correlation (*R* ^2^ = 0.5607) and a significantly reduced slope (0.6492). This disparity suggests that vaccine‐induced immunity is particularly effective in limiting the production of viable progeny during natural exposure. Consequently, conventional qPCR significantly overestimated viral risk in vaccinated fish exposed via IM, whereas vqPCR accurately reflected the suppressed infectivity (Figure 10D).

### 3.4 Comparative Analysis of Viral Loads in Deceased and Surviving Fish

**FIGURE 11:**
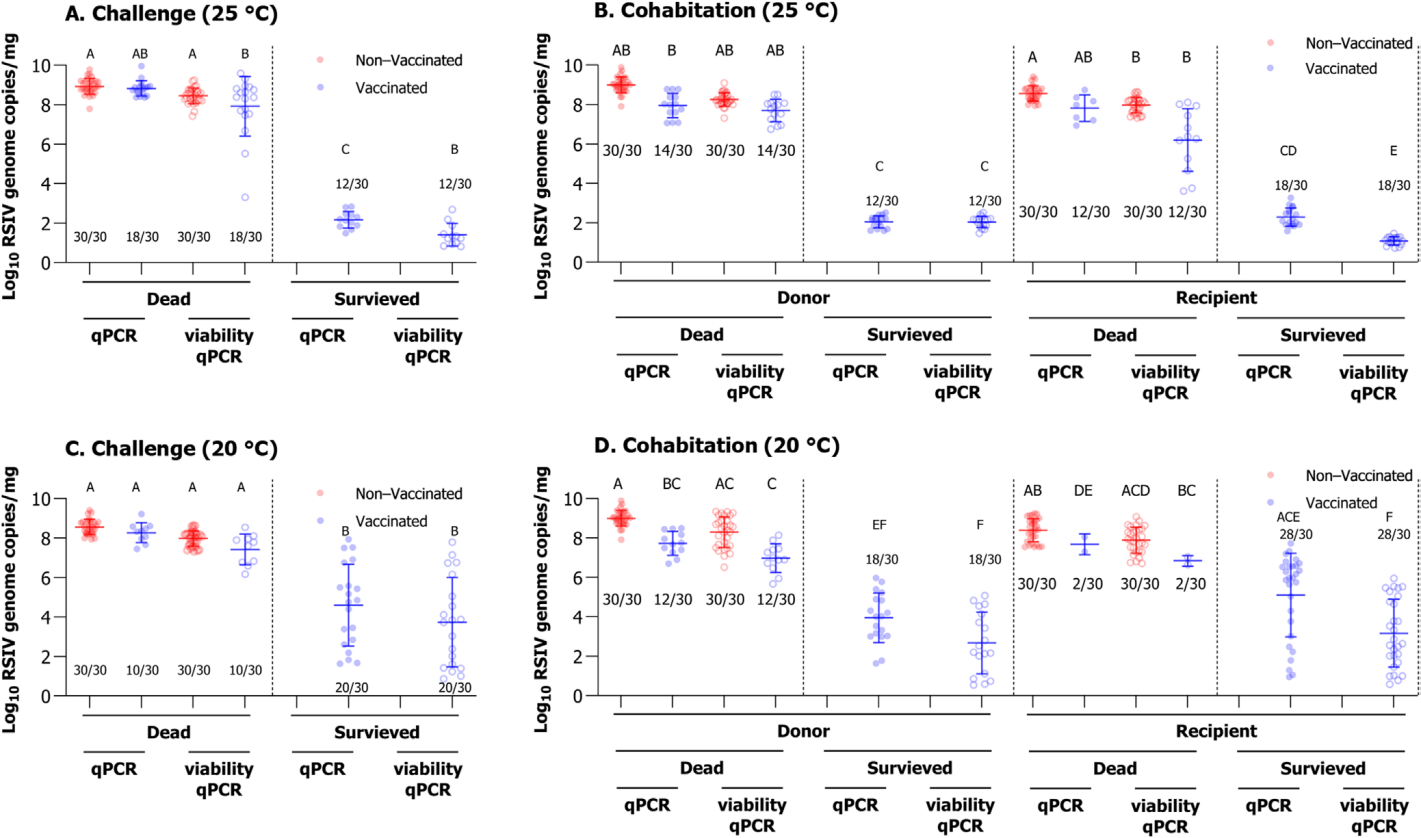
Distinct viral burden profiles distinguishing deceased and surviving rock bream ( *Oplegnathus fasciatus* ) following intraperitoneal (IP) red sea bream iridovirus (RSIV) challenge. (A, B) Direct IP challenge models at 25°C (A) and 20°C (B). (C, D) Cohabitation models at 25°C (C) and 20°C (D). Comparative quantification. Viral copy levels were compared between deceased and surviving fish within each group. The figure depicts clear demarcation where deceased fish consistently exhibited high viral loads (mean > 10^8^ copies/mg), whereas surviving fish maintained significantly suppressed viral burdens, indicating a potential lethal threshold. All values are represented as Log_10_ (total/infectious) viral copies per milligram of spleen. Statistical significance was determined using one‐way analysis of variance with Dunnett's test (*p < 0.05).

**FIGURE 12:**
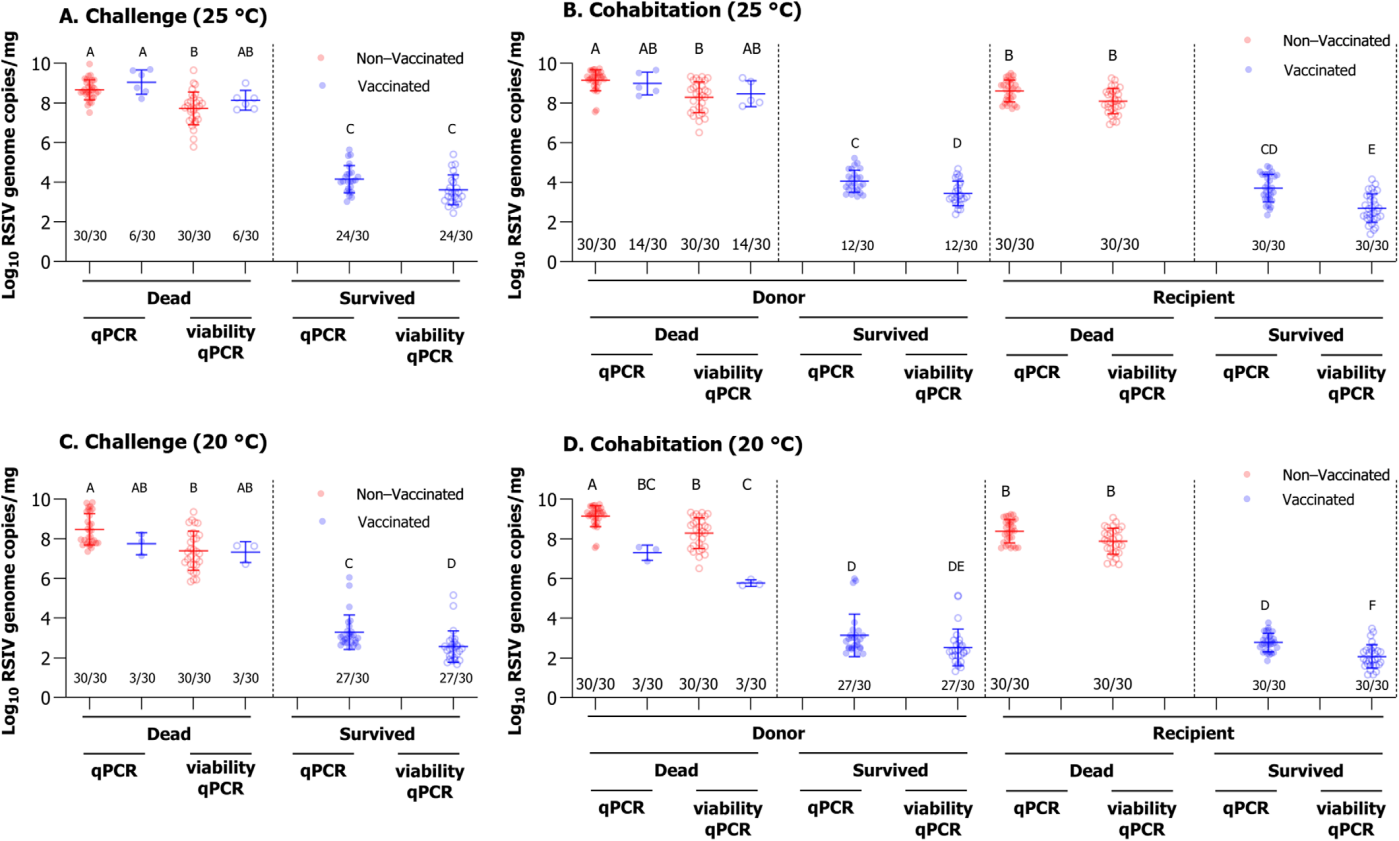
Distinct viral burden profiles distinguishing deceased and surviving rock bream following red sea bream iridovirus (RSIV) immersion challenge. (A, B) Immersion challenge models at 25°C (A) and 20°C (B). (C, D) Cohabitation models at 25°C (C) and 20°C (D). Comparative quantification. Viral copy levels were compared between deceased and surviving fish within each group. Similar to the IP model, deceased individuals exhibited consistently high viral loads regardless of the infection route, whereas survivors maintained sublethal viral levels. All values are represented as Log_10_ (total/infectious) viral copies per milligram of spleen. Statistical significance was determined using one‐way analysis of variance with Dunnett's test (*p < 0.05).

We observed a strong inverse correlation between the infectious viral burden and host survival across various treatments and temperatures (Figures 11 and 12). Deceased fish, predominantly from non‐vaccinated groups, consistently harboured high total and infectious viral copy numbers (mean > 10^8^ copies/mg). Survivors, primarily from vaccinated groups, exhibited significantly lower viral loads than their deceased counterparts within each corresponding group (*p* < 0.05). The mean viral load in most surviving groups remained below approximately 10^5^ copies/mg for total virus and 10^4^ copies/mg for infectious virus. This marked divergence reveals a mechanism of vaccine‐induced protection in viral load profiles, whereby viral replication is suppressed to sublethal levels.

## 4 Discussion

Understanding how vaccination influences RSIV transmission dynamics is essential for effective disease control in rock bream aquaculture. Historically, vaccine assessments have heavily relied on survival metrics and conventional qPCR or PCR methodologies, both of which fail to differentiate between infectious virions and noninfectious genetic debris (Kwon et al. 2020a; Min et al. 2024). Consequently, the precise mechanisms by which vaccination modulates viral shedding kinetics and epidemiological risks have remained poorly understood. In this study, we conducted a comprehensive investigation of RSIVD dynamics through a dual‐quantification framework that integrates FeCl_3_‐based iron flocculation with vqPCR. By applying this approach to both IP and IM challenge models, we distinguished active viral replication from inert genomic residues, providing novel insights into how vaccination induces “functional sterilization” and fundamentally alters the horizontal transmission dynamics of RSIV.

In the present study, water temperature significantly affected the temporal dynamics of RSIV replication, consistent with previous epidemiological studies. For example, both Kim et al. (2022) and Jun et al. (2009) reported that RSIV pathogenicity is highly temperature‐dependent, with rapid viral replication at high temperatures (25°C) and markedly suppressed replication at lower temperatures (20°C). Our findings successfully recapitulate these established characteristics: viral replication and disease progression occurred rapidly at 25°C but were delayed at 20°C. Despite these pronounced differences in viral kinetics, the vaccinated group consistently achieved high RPS regardless of water temperature. These results confirm two key points: first, our experimental model faithfully reproduces the natural course of RSIV infection; second, the vaccine confers robust protection against RSIVD under varying thermal regimes.

A fundamental challenge in vaccine evaluation arises from the divergence of mechanisms between artificial and natural infection routes. IP injection delivers the virus directly into systemic circulation, completely bypassing the mucosal barriers that serve as the primary line of defense in aquatic species (Salinas et al. 2011). In contrast, waterborne exposure requires the pathogen to breach mucosal surfaces, such as the skin, gills, or gut, before establishing a systemic infection. This mechanistic distinction holds crucial immunological implications; unlike IP injection, IM vaccination effectively elicits mucosal IgT responses critical for blocking pathogen entry at barrier surfaces (Xu et al. 2013; Munangandu et al. 2015). These findings suggest route‐specific immune involvement, although further immunological analyses are required to confirm this hypothesis. Notably, our results reflect this biological distinction. Specifically, in the IM model, peak viral loads were delayed by 11 days compared to the IP model, and the cohabitation challenge demonstrated distinct viral dynamics characteristic of continuous natural exposure rather than acute challenge. Given that disease transmission in real‐world aquaculture settings occurs primarily through waterborne routes, relying solely on injection models underestimates the critical role of mucosal immunity. Therefore, we propose that validating vaccine efficacy using experimental designs that closely mimic natural exposure pathways provides a more accurate estimate of practical protection under field scenarios.

A critical concern with inactivated vaccines is incomplete inactivation or reversion to virulence. In our safety assessment, we detected transient viral DNA signals (up to 10^4.4^ copies/mg) in the vaccinated control group during the initial 10 d post‐vaccination. Therefore, reliance on conventional qPCR could yield the misleading conclusion that vaccine strains persist or replicate. However, vqPCR analysis in our study confirmed that the detection of infectious particles was significantly suppressed in vaccinated control groups. These results suggest that the inactivated viral antigens were effectively recognized and processed by the host immune system, leading to the degradation of viral particles into noninfectious genomic debris (Leiva‐Rebollo et al. 2024). Therefore, the PMAxx‐vqPCR platform proved essential for differentiating these noninfectious immune clearance products from active viral risks, offering a precise interpretation of vaccine safety.

We also observed method‐dependent divergence in viral detection, highlighting the superior diagnostic precision of vqPCR over conventional qPCR. In the IP injection model, direct introduction of a high viral inoculum appeared to overwhelm immediate host defenses, resulting in a fixed ratio of infectious virions to total DNA, regardless of vaccination status (*R* ^2^ > 0.8, slopes ≈1.0). Under these conditions, conventional qPCR served as a sufficient proxy for infectivity. However, the IM challenge model revealed the limitations of conventional qPCR in scenarios mimicking natural infection. In this context, gradual viral infiltration allows vaccine‐induced immune responses to compromise viral structural integrity before systemic dissemination occurs. Consequently, while conventional qPCR detected persistent genomic residues, vqPCR revealed a distinct “decoupling” between viral load and actual infectivity in vaccinated fish (*R* ^2^ = 0.56; slope = 0.6492). Even when genomic loads in vaccinated survivors reached levels corresponding to Grade 2 (10^2.7^–10^4.6^ copies/mg) or Grade 3 severity, the actual infectious titers remained suppressed based on severity grade thresholds (Kim, Kim, et al. 2024). This statistical evidence confirms that vaccination induces “functional sterilization,” delaying viral replication within infected hosts and effectively preventing the assembly of viable virus particles by enhancing immune responses. As a result, the host immune system degrades viral components before they can mature into infectious virions, resulting in high survival rates despite detectable viral DNA persistence. Notably, this reduction in viral infectivity translates directly into epidemiological safety. For example, in our cohabitation experiments, conventional qPCR detected residual viral DNA shedding from vaccinated donors, which might be interpreted as a potential transmission risk. However, to accurately assess this risk, it is essential to contextualize viral shedding levels against the minimum infectious dose (MID) for waterborne transmission, which has been established at approximately 10^3^–10^4^ copies/L in previous epidemiological models (Kawato et al. 2023). Our vqPCR analysis revealed that the shedding of infectious virions from vaccinated donors was negligible and consistently remained well below this MID threshold. This finding explains why naïve recipients cohabiting with vaccinated donors exhibited 90%–100% survival, whereas those exposed to non‐vaccinated donors experienced 100% mortality. Collectively, these results confirm that vaccination affects disease dynamics through a dual mechanism: it suppresses viral replication within the infected host while significantly reducing the release of viable progeny below the critical threshold required for transmission, effectively securing herd immunity.

In conclusion, this study confirms that vaccination effectively suppresses viral replication and the assembly of infectious particles within infected individuals, while also mitigating the risk of horizontal transmission by reducing the shedding of viable pathogens. These findings support the adoption of viability‐based assessment strategies for RSIV monitoring. Detection of viral DNA in the absence of infectious particles (qPCR‐positive/vqPCR‐negative) should be interpreted as evidence of successful immune clearance rather than an active biosecurity threat. Therefore, we recommend integrating environmental DNA surveillance with vqPCR‐based viability assessment to avoid unnecessary therapeutic interventions. Nevertheless, future longitudinal field studies are essential to validate these findings under the complex stressors of commercial aquaculture. Ultimately, establishing a viability‐based model provides a scientific foundation for optimizing vaccination protocols and refining risk management standards in the industry.

## Author Contributions

**Sung‐Bin Moon:** conceptualization, methodology, validation, formal analysis, investigation, data curation, visualization, writing – original draft, writing – review and editing. **Gyoungsik Kang:** conceptualization, methodology, validation, formal analysis and investigation. **HyeongJin Roh:** conceptualization, methodology, validation, formal analysis and investigation. **Yoonhang Lee:** conceptualization, methodology, validation, formal analysis and investigation. **Min‐Jae Kim:** methodology, formal analysis and investigation. **Min‐Young Sohn:** methodology, formal analysis and investigation. **Ha‐Jeong Son:** methodology, formal analysis and investigation. **Chan‐Il Park:** conceptualization, funding acquisition, data curation, supervision, resources, writing, review and editing. **Kyung‐Ho Kim:** conceptualization, methodology, validation, formal analysis, investigation, data curation, visualization, writing the original draft, writing the review and editing, funding acquisition, supervision, resources.

## Funding

This work was supported by a New Faculty Research Support Grant from Gyeongsang National University in 2025 (GNU‐NFRSG‐0062) and the Basic Science Research Program through the National Research Foundation of Korea (NRF) funded by the Ministry of Education of the Republic of Korea (RS‐2023‐00272416).

## Ethics Statement

The Animal Care and Use Committee Office of Gyeongsang National University approved the animal study under the institutional approval code GNU‐241204‐E0241.

## Conflicts of Interest

The authors declare no conflicts of interest.

## Data Availability

Data will be made available upon reasonable request from the corresponding authors.
